# Supplementary material for: Vitamin C Suppresses Pancreatic Carcinogenesis through the Inhibition of Both Glucose Metabolism and Wnt Signaling
Source: Int J Mol Sci. 2022 Oct 14;23(20):12249. doi: 10.3390/ijms232012249 (PMC9603812; doi:10.3390/ijms232012249)
Supplement: Supplementary file 1 [file ijms-23-12249-s001.zip › ijms-1824683-supplementary.pdf]

Supplemental Information

Vitamin C Suppresses Pancreatic Carcinogenesis through the Inhibition of Both Glucose Metabolism and Wnt Signaling

Ji Hye Kim †, Sein Hwang †, Ji-Hye Lee, Se Seul Im and Jaekyoung Son *

Department of Biomedical Sciences, Asan Medical Center, AMIST, University of Ulsan College of Medicine, Seoul 05505, South Korea

* Correspondence: jaekson@amc.seoul.kr

† These authors contributed equally to this work.

Supplementary Figure S1

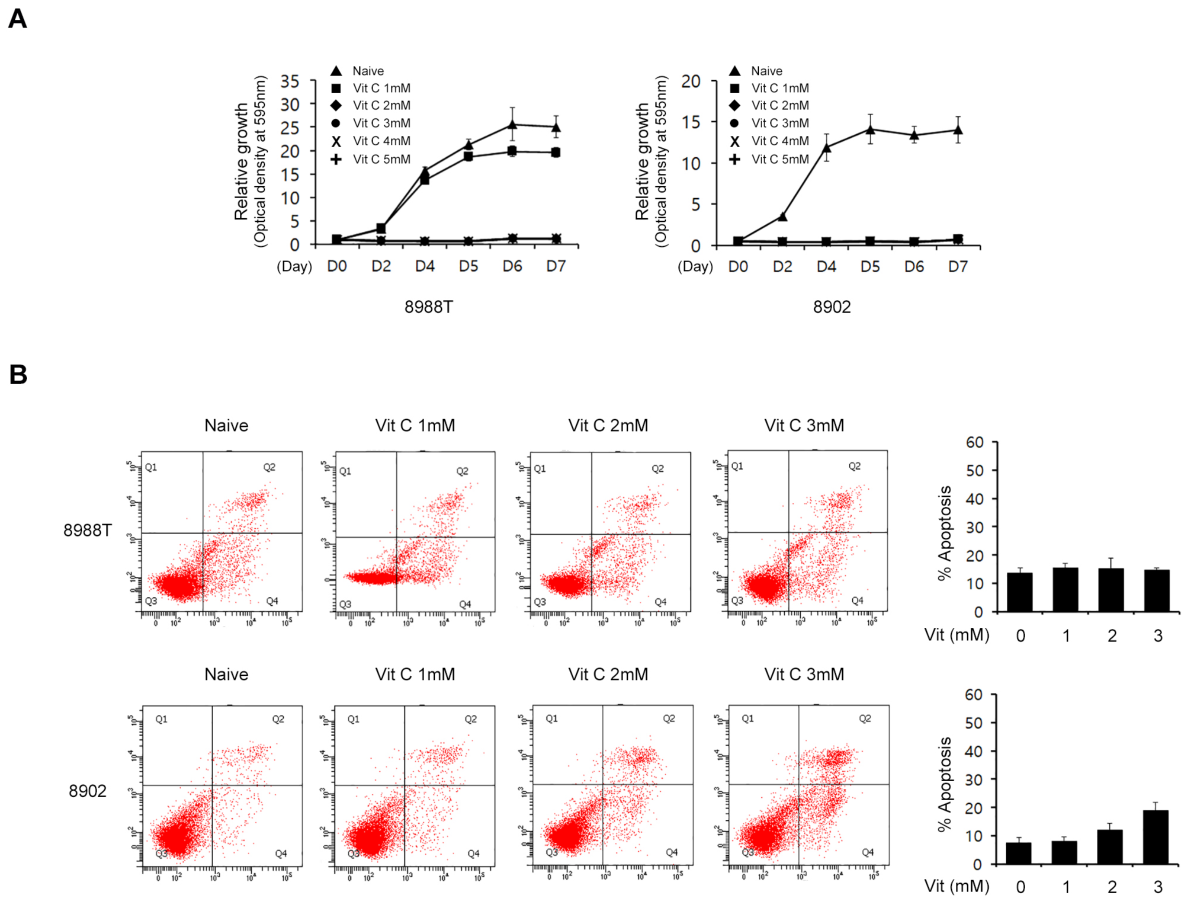

Supplementary Figure S1. Anti-tumor effects of vitamin C in PDAC cells. (**A**) Growth assay for PDAC cells treated with vitamin C for the indicated days. (**B**) PDAC cells were treated with vitamin C for 24 h and assayed for apoptotic cell death by annexin V/PI staining and flow cytometry. Error bars represent s.d. of triplicate wells from a representative experiments.

Supplementary Figure S2

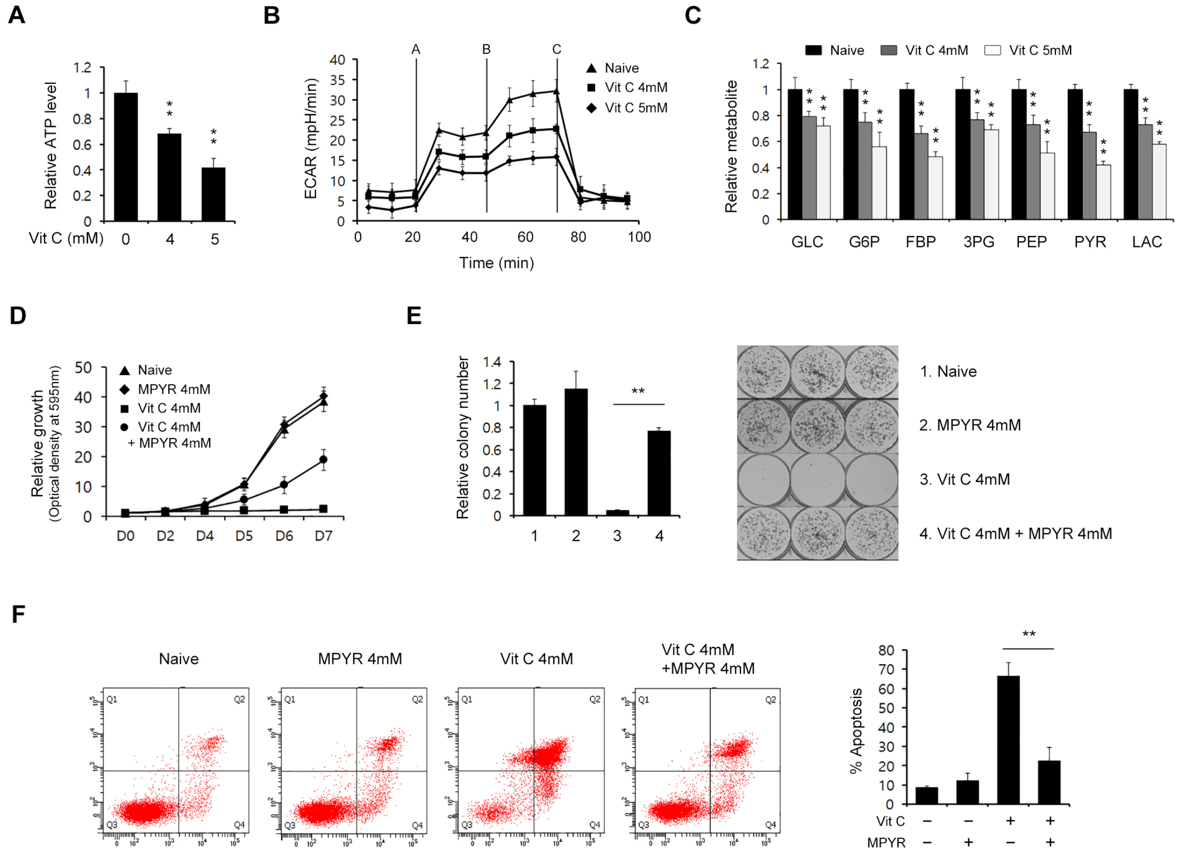

Supplementary Figure S2. Effect of high-dose vitamin C on glycolytic flux. (**A**) 8902 cells were treated with vitamin C (4 or 5 mM) for 24 h and assayed for intracellular ATP. (**B**) 8902 cells were treated with vitamin C (4 or 5 mM) for 24 h and the extracellular acidification rate was measured. (**C**) 8902 cells were treated with vitamin C (4 or 5 mM) for 24 h and analyzed for glycolysis metabolite pools via LC/MS-MS. (**D**) Cell growth assay for 8902 cells treated with vitamin C (4 mM) for 8 days with or without methyl-pyruvate (4 mM). (**E**) Clonogenic assay for 8902 cells treated with vitamin C (4 mM) for 8 days with or without methyl-pyruvate (4 mM). (**F**) 8902 cells were treated with vitamin C (4 mM) for 8 days with or without methyl-pyruvate (4 mM) and assayed for apoptotic cell death by annexin V/PI staining and flow cytometry. Error bars represent s.d. of triplicate wells from a representative experiment; ** $p < 0.01$.
